# Allogenic Adipose Tissue-Derived Mesenchymal Stem Cells Ameliorate Acute Hepatic Injury in Dogs

**DOI:** 10.1155/2017/3892514

**Published:** 2017-12-28

**Authors:** Takahiro Teshima, Hirotaka Matsumoto, Masaki Michishita, Akito Matsuoka, Maika Shiba, Tomokazu Nagashima, Hidekazu Koyama

**Affiliations:** ^1^Laboratory of Veterinary Internal Medicine, Department of Veterinary Clinical Medicine, School of Veterinary Medicine, Faculty of Veterinary Science, Nippon Veterinary and Life Science University, 1-7-1 Kyonan-cho, Musashino-shi, Tokyo 180-8602, Japan; ^2^Laboratory of Veterinary Pathology, Department of Veterinary Pathobiology, School of Veterinary Medicine, Faculty of Veterinary Science, Nippon Veterinary and Life Science University, 1-7-1 Kyonan-cho, Musashino-shi, Tokyo 180-8602, Japan

## Abstract

Adipose tissue-derived mesenchymal stem cells (AT-MSCs) are an attractive source for cell-based therapy of some diseases, including acute and chronic liver failure, in not only human medicine but also veterinary medicine. However, in veterinary medicine, no studies have reported the effects of AT-MSCs on liver injury in dogs. The purpose of this study was to investigate the effects of allogenic AT-MSCs on acute liver injury by carbon tetrachloride in dogs and to compare the therapeutic effects of AT-MSCs transplanted via the peripheral vein (PV) or splenic vein (SV). After transplantation of AT-MSCs through the PV or SV, serum liver enzymes were decreased significantly, and SV injection was more effective compared with PV injection. By comparing the number of engrafted AT-MSCs in the liver, SV injection was significantly more effective than PV injection. mRNA expression levels of proinflammatory cytokines, such as IL-1, IL-6, IL-8, and IFN*γ*, in the liver were decreased significantly, but those of anti-inflammatory cytokines, such as IL-4 and IL-10, HGF, and VEGFA, were significantly increased after the first AT-MSC injection. These findings suggest that allogenic AT-MSCs injected via the PV or SV ameliorate acute hepatic injury in dogs, and AT-MSCs injected via the SV provide more effective improvement.

## 1. Introduction

Recently, mesenchymal stem cell (MSC) transplantation has been studied as a therapeutic approach for various diseases. The common tissue origins of MSCs are bone marrow and adipose tissue. Reports have shown that MSCs are useful for improvement of acute and chronic liver failure in animal models [1–3]. Moreover, some reports have described that bone marrow-derived MSCs (BM-MSCs) exert therapeutic effects in patients with liver cirrhosis and liver failure [4–6]. However, the amount of available bone marrow is usually insufficient and the acquirement procedure is invasive. Conversely, adipose tissue-derived MSCs (AT-MSCs) have similar biological properties to BM-MSCs [7] and are abundantly present in the body. Thus, AT-MSCs can be harvested repeatedly by simple and minimally invasive procedures.

In veterinary medicine, AT-MSCs of some species, including dogs, cats, and horses, have been investigated by isolation and characterization, and clinical trials of AT-MSCs have been reported for some diseases such as osteoarthritis, inflammatory bowel disease, chronic kidney disease, and tendonitis [8, 9]. However, no reports have evaluated the effects of AT-MSCs on liver diseases. Therefore, this study aimed to determine the effects of allogenic canine AT-MSCs on acute liver injury induced by carbon tetrachloride (CCl_4_) and the therapeutic effects of AT-MSCs injected via different transplantation routes.

## 2. Materials and Methods

### 2.1. Animal Models

**Figure 1:**
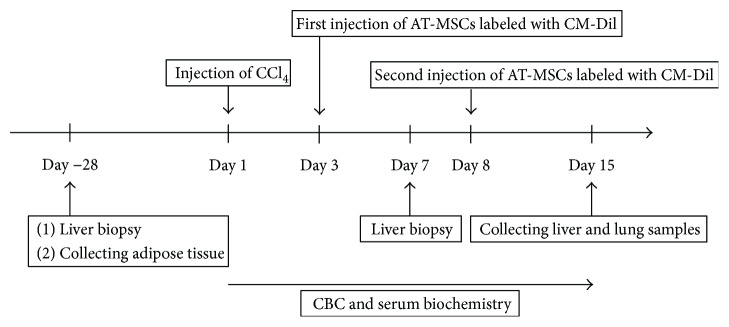
Experimental design. AT-MSCs: adipose tissue-derived stem cells; CBC: complete blood count; CCl4: carbon tetrachloride.

Nine healthy beagles (nine intact males; mean age: 1.5 years; mean body weight: 12.2 kg) were used in this study. Dogs were handled in accordance with the animal care guidelines of the Institute of Laboratory Animal Resources, Nippon Veterinary and Life Science University, Japan. The dogs were assigned to control, peripheral vein administration (PV), or splenic vein administration (SV) groups (*n* = 3 dogs/group). The Institutional Animal Care and Use Committee of Nippon Veterinary and Life Science University approved the experimental design (S27S-13). The experimental design of the study is summarized in Figure 1.

### 2.2. Induction of Acute Hepatic Injury

Experimental acute hepatic injury was induced by the injection of CCl_4_. On day 1, dogs were injected with 20 *μ*g/kg medetomidine (Nippon Zenyaku Kogyo) intravenously for sedation. Then, the dogs were injected intraperitoneally with CCl_4_ (WAKO; diluted at 1 : 1 in corn oil) at a dose of 0.25 ml/kg under ultrasound guidance.

### 2.3. Canine AT-MSCs

#### 2.3.1. Isolation and Culture of Canine AT-MSCs

Adipose tissue was aseptically collected from falciform ligament fat of an anaesthetized dog in the control group at 4 weeks before induction of acute liver injury. The tissue was washed extensively in PBS, minced, and digested with collagenase type I (Sigma-Aldrich) at 37°C for 45 min with intermittent shaking. After washing with PBS and centrifuging, the pellets containing the stromal vascular fraction were resuspended, filtered through a 100 *μ*m nylon mesh, and incubated overnight in high glucose Dulbecco's Modified Eagle's Medium (H-DMEM) supplemented with 10% fetal bovine serum (FBS; Nichirei Bioscience) and a 1% antibiotic-antimycotic solution (Thermo Fisher Scientific) in a humidified atmosphere with 5% CO_2_ at 37°C. Unattached cells were removed by changing the medium, and the attached cells were washed twice with PBS. Thereafter, the medium was replaced every 3-4 days. At 80–90% confluence, the cells were detached with trypsin-EDTA solution (Sigma-Aldrich) and passaged repeatedly.

#### 2.3.2. Characterization of AT-MSC Surface Markers

Passage 2 AT-MSCs were analyzed by flow cytometry. The cells were placed in fluorescence-activated cell sorting (FACS) tubes (BD Biosciences; 2 × 10^5^ cells/tube), washed with FACS buffer (PBS containing 2% FBS), blocking Fc receptors with canine Fc receptor binding inhibitor (Thermo Fisher Scientific), and then incubated with the following fluorescein- (FITC-) or phycoerythrin- (PE-) conjugated antibodies: anti-CD14-FITC (BD PharMingen), anti-CD29-PE (BioLegend), anti-CD34-PE (R&D Systems), anti-CD44-PE (BioLegend), anti-CD45-FITC (eBioscience), and anti-CD90-PE (eBioscience) or their respective isotype controls listed in Table 1. The cells were washed twice with FACS buffer and resuspended in 500 *μ*l FACS buffer. Fluorescence was evaluated by flow cytometry in a FACSCalibur instrument (BD Biosciences). Data were analyzed using WinMDI 2.9 analysis software.

#### 2.3.3. Differentiation Assay

For osteogenic differentiation, passage 2 AT-MSCs were seeded on 6-well plates (5.0 × 10^3^ cells/cm^2^) and incubated in H-DMEM supplemented with 10% FBS and 1% antibiotic-antimycotic solution for 24 h. The medium was then changed to osteogenic medium (Cell Applications) [10]. The medium was changed twice weekly. For osteogenic analysis, mineral deposits were quantitatively analyzed by von Kossa staining after 21 days.

For adipogenic differentiation, passage 2 AT-MSCs were seeded on 6-well plates (8 × 10^3^ cells/cm^2^) and cultured in H-DMEM supplemented with 10% FBS and 1% antibiotic-antimycotic solution until confluency. Then, the medium was changed to canine adipocyte differentiation medium (Cell Applications). The medium was changed twice weekly. Adipogenesis was analyzed by Oil Red O staining after 21 days.

### 2.4. AT-MSC Transplantation

**Figure 2:**
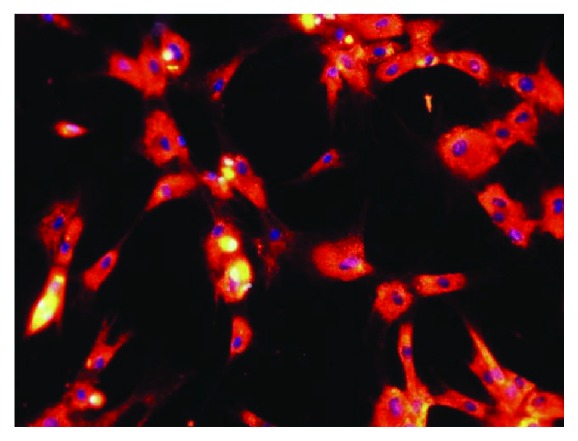
AT-MSCs labeled with CM-Dil before injection. Approximately 95% of AT-MSCs were labeled with CM-Dil according to the manufacturer's procedure. AT-MSCs were mounted in VECTASHIELD-mounting medium with DAPI (Vector Laboratories) as a nuclear counterstain.

AT-MSCs were labeled with CellTracker CM-DiI (Thermo Fisher Scientific) before injection according to the manufacturer's instructions and suspended in 0.9% saline (Figure 2). The PV group was injected with labeled AT-MSCs via the cephalic vein at a dose of 2 × 10^6^ cells/kg. The SV group was injected with labeled AT-MSCs via the splenic vein at a dose of 2 × 10^6^ cells/kg under ultrasound guidance. On day 3, PV and SV groups were injected with passage 3 AT-MSCs and then injected with passage 4 AT-MSCs on day 8. The control group was injected with the same volume of 0.9% saline via the cephalic vein. All dogs were injected with labeled AT-MSCs or 0.9% saline after sedation with medetomidine (20 *μ*g/kg by intravenous injection).

### 2.5. Evaluation of White Blood Cell Counts and Liver Enzymes Activities

To evaluate the acute hepatic injury, levels of liver enzymes and white blood cells were measured every day during the study. Blood samples were obtained from the jugular vein and transferred to tubes containing EDTA and normal serum. Sera were separated by centrifugation at 4°C for 15 min and stored at −80°C until assayed.

White blood cell counts were obtained using a hematology analyzer (SYS-POCH-100i), and levels of serum alanine aminotransferase (ALT), aspartate aminotransferase (AST), and alkaline phosphatase (ALP) were measured using an automated clinical chemistry analyzer (Fuji Dry-Chem).

### 2.6. Laparoscopic Liver Biopsy

All dogs were subjected to a laparoscopic liver biopsy on day 7. Dogs were medicated and anesthetized with butorphanol tartrate (Meiji Seika Pharma; 0.2 mg/kg by subcutaneous injection) and propofol (Mylan; 7 mg/kg by intravenous injection). Anesthesia was maintained with isoflurane (DS Pharma Animal Health) throughout the procedure. Under sterile conditions, dogs were positioned in dorsal recumbency. A trocar cannula was inserted into the ventral midline at 1-2 cm caudal to the umbilicus by the Hasson technique [11]. The peritoneal cavity was distended with carbon dioxide to a maximum pressure of 12 mm Hg, and a trocar cannula was placed to allow insertion of a 5 mm cup biopsy instrument into the abdomen. Liver samples were obtained by grasping the hepatic parenchyma with a cup biopsy instrument and then pulling the tissue sample out with gentle traction.

### 2.7. Histological Evaluation

On day 15, each dog was euthanized by an intravenous overdose of pentobarbital after collecting blood samples. Tissue samples were obtained from each liver lobe and caudal lobe of the right lung. Samples on days 7 and 15 were fixed in 4% paraformaldehyde for histological examination or stored at −80°C for RNA extraction. Liver samples from each lobe for histological examination were dehydrated and embedded in paraffin and then sectioned at 4 *μ*m thicknesses. The sections were stained with hematoxylin and eosin (H&E) and Masson's trichrome (MT).

Quantification of tissue fibrosis was based on 5 fields taken at random from each lobe slide. The percentage of fibrosis area was calculated in each image using the Image J software version 1.45 (https://imagej.nih.gov/ij/).

### 2.8. Immunohistochemical Staining

Immunohistochemistry was performed on serial sections using the labeled streptavidin-biotin method with the primary antibodies listed in Table 2. The sections were treated with 0.03% H_2_O_2_ in 33% methanol at room temperature for 30 min to block endogenous peroxidase and then underwent antigen retrieval treatment (Table 2), followed by an incubation in a 4% milk solution at room temperature for 30 min. Finally, the reaction to each antigen was visualized by the addition of 3,3′-diaminobenzidine tetrahydrochloride chromogen and counterstaining with hematoxylin.

For quantification of inflammatory cell infiltration, myeloperoxidase and CD163 positive cells were counted in 5 fields taken at random from each slide.

### 2.9. Evaluation of the Number of Engrafted AT-MSCs

Liver and lung samples were fixed in 4% paraformaldehyde at 4°C overnight. For cryosectioning, samples were washed in 30% sucrose, embedded in OCT compound (Sakura Finetek), and frozen. Sections were cut at 8 *μ*m thicknesses. AT-MSCs labeled with CellTracker CM-DiI were counted under a microscope in five random fields for each liver and lung sample.

### 2.10. Real-Time Quantitative PCR Analyses of Cytokine Expression

Cytokines expression in the liver samples was determined by real-time quantitative PCR. Total RNA was extracted using a TRIzol Plus RNA Purification Kit according to the manufacturer's instructions (Thermo Fisher Scientific). cDNA was synthesized from 1 *μ*g total RNA using random primers and the GoScript Reverse Transcriptase system (Promega), according to the manufacturer's instructions. Real-time RT-PCR analyses were performed using SYBR Green Real-time PCR Master Mix (Promega) to determine the mRNA levels of interleukin- (IL-) 1*β*, IL-4, IL-6, IL-8, IL-10, tumor necrosis factor-*α* (TNF*α*), interferon gamma (IFN*γ*), hepatocyte growth factor (HGF), and vascular endothelial growth factor (VEGF) A. The primer sequences are listed in Table 3. Amplification conditions were 95°C for 2 min, followed by 40 cycles of 95°C for 15 sec and 60°C for 60 sec. After 40 cycles, a dissociation curve was generated to verify the specificity of each primer. All reactions were performed in duplicate. Expression levels of target genes were normalized to the level of glyceraldehyde 3-phosphate dehydrogenase and quantified by the ΔΔCt method.

### 2.11. Statistical Analysis

All data are presented as the mean ± standard deviation. Differences between two groups were compared with Welch's *t*-test. Differences among multiple groups were assessed by one-way or two-way analysis of variance and then differences were compared using the Tukey-Kramer post hoc test. A value of *P* < 0.05 was considered statistically significant. Statistical analyses were performed using Excel 2010 with add-in software Statcel 3.

## 3. Results

### 3.1. Characterization of AT-MSCs

**Figure 3:**
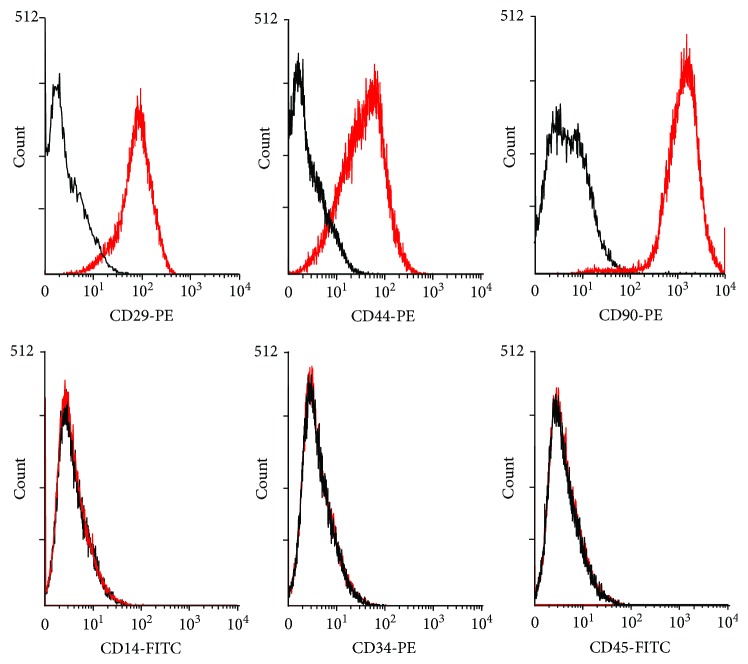
Results of flow cytometry. Black lines represent isotype controls and red lines indicate AT-MSCs. AT-MSCs were positive for CD29, CD44, and CD90 and negative for CD14, CD34, and CD45.

**Figure 4:**
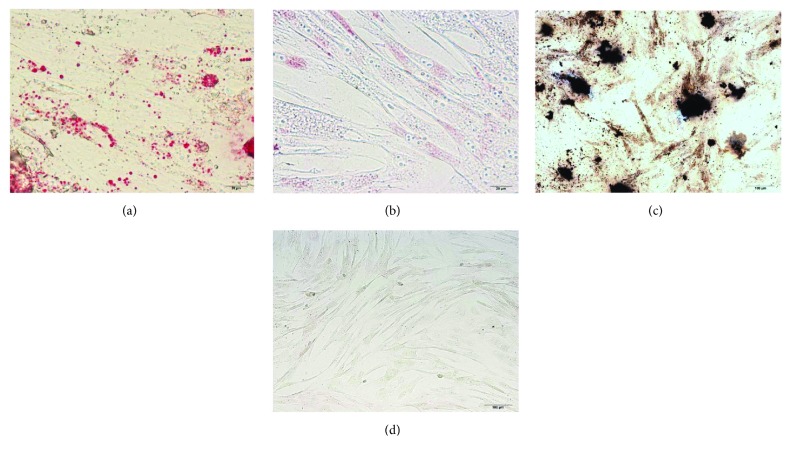
Multilineage differentiation of canine AT-MSCs. (a) Adipogenic differentiation was identified by Oil Red O staining. (b) Oil Red O staining of control undifferentiated cells. (c) Osteogenic differentiation was identified by von Kossa staining. (d) von Kossa staining of control undifferentiated cells.

AT-MSCs were successfully cultured and expanded. The majority of the cells expressed the established MSC markers CD29 (95.2%), CD44 (91.6%), and CD90 (99.4%), and very few expressed CD14 (0.8%), CD34 (0.6%), or CD45 (0.7%) (Figure 3). The AT-MSCs exhibited multilineage plasticity as demonstrated by their potential for adipogenic and osteogenic differentiation, compared with undifferentiated cells (Figure 4).

### 3.2. Effects of AT-MSC Transplantation on Levels of Liver Enzymes and White Blood Cells

**Figure 5:**
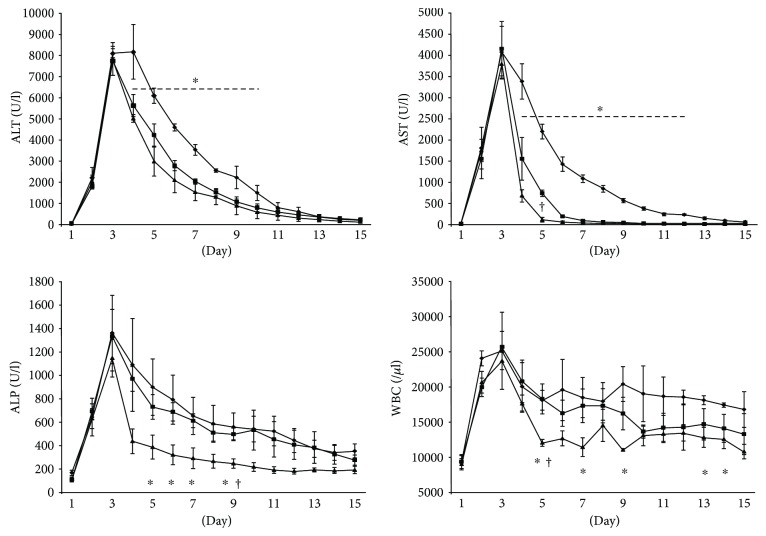
Serum levels of ALT, AST, and ALP and white blood cell counts. Data are expressed as the mean ± standard deviation. ◆: control group; ■: PV group; ▲: SV group. ALT levels in PV and SV groups were significantly decreased from day 4 to day 10 compared with the control. AST levels in PV and SV groups were significantly decreased from day 4 to day 12 compared with the control. ^∗^*P* < 0.05 versus control group; ^†^*P* < 0.05 versus PV group.

Before injection with CCl_4_ (Days 14 and 7), it was confirmed that physical examination, complete blood count, and blood chemistry were normal in the control group resected in the falciform ligament fat for collecting the adipose tissue. Therefore, resection of falciform ligament fat was not affected in this study from day 1. Sequential changes in the levels of liver enzymes and white blood cells are shown in Figure 5. After inducing acute hepatic injury, levels of liver enzymes and white blood cells were increased markedly. ALT in PV and SV groups was decreased the next day after injecting the first AT-MSCs, but that in the control group was decreased from day 5. After the first injection of AT-MSCs, ALT was significantly decreased in the PV and SV group at day 4 (PV group: 5635 ± 650 U/L; SV: group 5023 ± 223 U/L) compared with the control group (8186 ± 1576 U/L) (*P* < 0.05) from day 4. AST was also significantly decreased in the PV and SV groups compared with the control group at day 4 (control group: 3388 ± 511 U/L; PV group: 1565 ± 611 U/L; SV group: 695 ± 175 U/L). AST in the SV group was also significantly decreased compared with the PV group at day 5 (131 ± 65 U/L versus 753 ± 92 U/L) (*P* < 0.05). After the first injection of AT-MSCs, ALP was significantly decreased in the SV group compared with the control group at day 5 (control group: 896 ± 295 U/L; PV group: 729 ± 128 U/L; SV group: 384 ± 124 U/L) (*P* < 0.05). ALP in the SV group was also significantly decreased compared with the PV group after the second injection of AT-MSCs on day 9 (243 ± 49 U/L versus 494 ± 65 U/L). White blood cells in the SV group were also decreased after the first injection of AT-MSCs, and those in the SV group were significantly decreased compared with control and PV groups on day 5 (control group: 18,133 ± 1724/*μ*l; PV group: 18,300 ± 2605/*μ*l; SV group: 12,066 ± 585/*μ*l) (*P* < 0.05).

### 3.3. Fibrosis and Inflammatory Cell Infiltration of Acute Hepatic Injury

**Figure 6:**
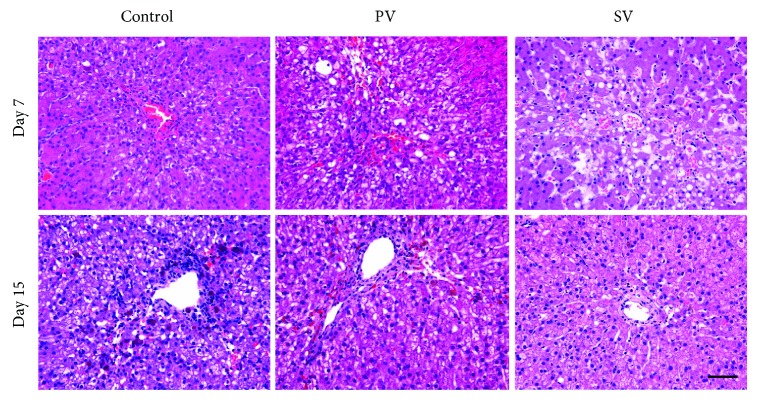
Histopathology of livers after AT-MSC injections. Inflammatory cell infiltration and necrosis in livers were observed at both of day 7 and 15. H&E staining. Bar = 50 *μ*m.

On day 7 and 15, inflammatory cell infiltration, hepatocellular degeneration and atrophy around the central vein, and hemorrhages were observed in all groups (Figure 6).

**Figure 7:**
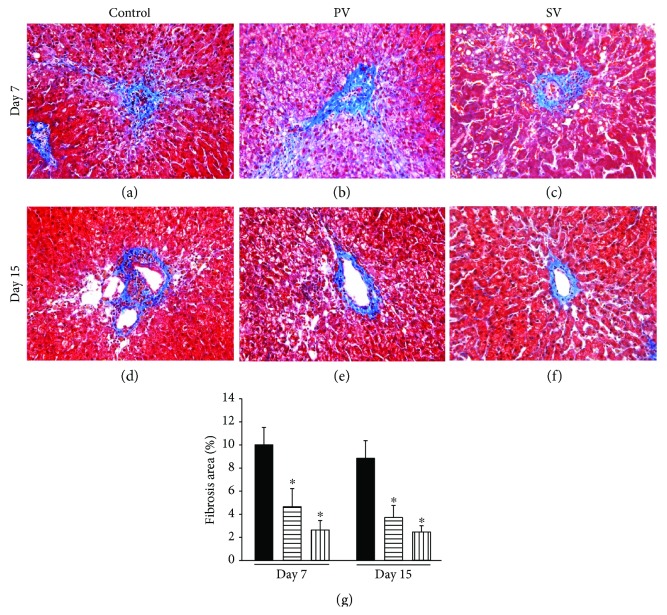
Hepatic fibrosis around the central vein at days 7 and 15. The degree of distribution of collagen deposition on day 7 (a, b, c) was higher than that on day 15 (d, e, f) in all groups. Comparison of the quantification of fibrosis area in liver sections (g). The fibrosis around the central vein in the control group was more apparent than that in the PV and SV groups. ■: control group; ▤: PV group; ▥: SV group. Data are expressed as the mean ± standard deviation. ^∗^*P* < 0.05. MT staining. Bar = 50 *μ*m.

Collagen deposition around the central vein was observed in all groups (Figure 7). The percentage of fibrosis area was significantly higher in the control group (10.0 ± 1.7%) compared with the PV and SV groups at day 7 (PV group: 4.6 ± 1.6%; SV group: 2.7 ± 0.8%) (*P* < 0.05). At day 15, the percentage of fibrosis area also significantly lower in the PV and SV groups compared with the control group (control group: 8.9 ± 1.4%; PV group: 3.7 ± 1.0%; SV group: 2.5 ± 0.5%) (*P* < 0.05), but there were no statistically significant differences between the day 7 and 15 in all groups.

**Figure 8:**
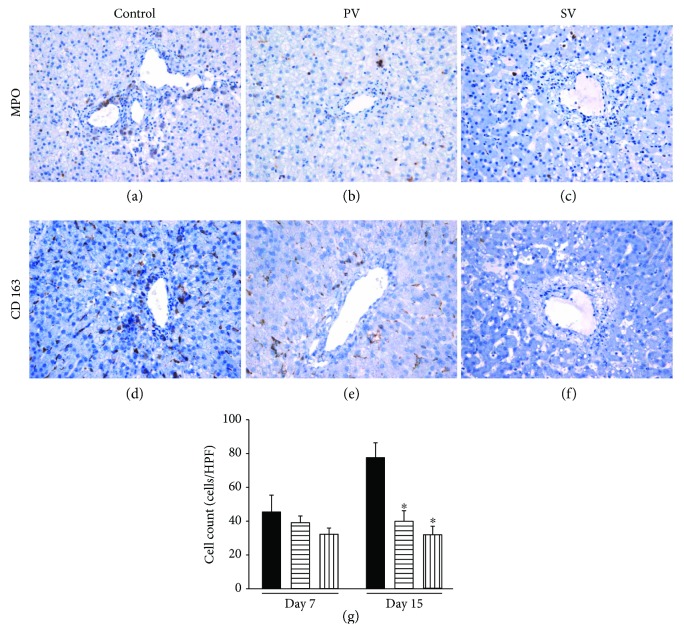
Inflammatory cell infiltration around the central vein in the liver. The myeloperoxidase (MPO) and CD163 positive cells at day 15 are shown (a–f). Comparison of the number of inflammatory cells in liver sections (g). ■: control group; ▤: PV group; ▥: SV group. Data are expressed as the mean ± standard deviation. ^∗^*P* < 0.05.

Neutrophils and macrophages were stained by myeloperoxidase (MPO) and CD163, respectively. The number of inflammatory cells (sum of MPO^+^ and CD163^+^ cells) was lower in the PV and SV group at day 7 (PV group: 39.0 ± 3.8 cells/HPF; SV group: 32.2 ± 3.5 cells/HPF) compared with the control group at day 7 (control group: 45.2 ± 10.2 cells/HPF), but there were no statistically significant differences among the groups. At day 15, the number of inflammatory cell was significantly higher in the control group (77.8 ± 8.4 cells/HPF) compared with the PV and SV group (PV group: 40.4 ± 6.1 cells/HPF; SV group: 32.0 ± 4.5 cells/HPF) (*P* < 0.05) (Figure 8).

### 3.4. Comparison of Engrafted AT-MSCs Numbers according to Route of Injection

**Figure 9:**
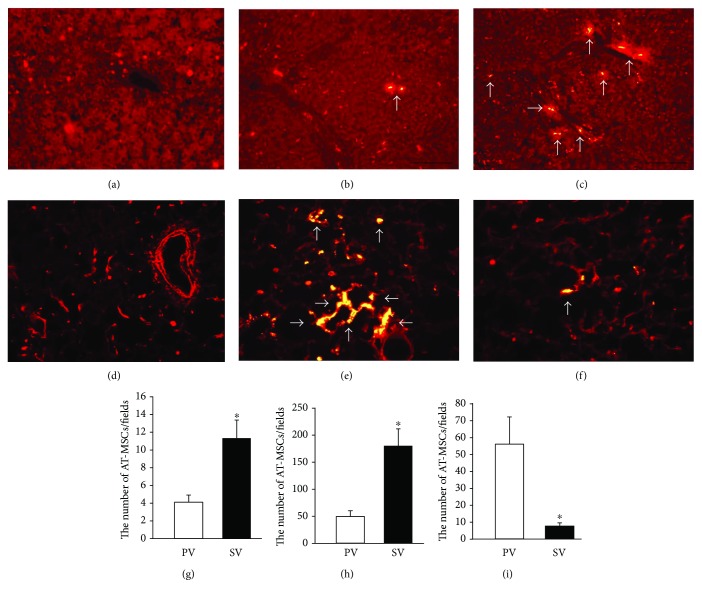
Engrafted AT-MSCs in liver and lung lobe sections. Liver (a) and lung (d) lobe sections of the control group. More AT-MSCs labeled with CM-DiI (white arrows) were found in liver lobe sections of the SV group (c) than in those of the PV group (b). In contrast, more AT-MSCs labeled with CM-DiI (white arrows) were found in lung lobe sections of the PV group (e) than in those of the SV group (f). Comparison of the number of AT-MSCs labeled with CM-DiI per field in liver sections at day 7 (g) and day 15 (h). Comparison of the number of AT-MSCs labeled with CM-DiI per field in lung sections at day 15 (i). Data are expressed as the mean ± standard deviation. ^∗^*P* < 0.05.

To evaluate the number of engrafted AT-MSCs based on the route of injection, AT-MSCs labeled with CellTracker CM-DiI were observed in liver and lung tissue samples (Figures 9(a)–9(d)). On day 7, before the second injection of AT-MSCs, the number of AT-MSCs in livers of the SV group (11.3 ± 2.1 cells) was 2.8-fold higher than that in the PV group (4.0 ± 0.8 cells) (*P* < 0.05) (Figure 9(e)). On day 15, the number of AT-MSCs in livers of the SV group (181.7 ± 31.6 cells) was 3.7-fold higher than that in the PV group (49.0 ± 11.0 cells) (*P* < 0.05) (Figure 9(f)). However, the number of engrafted AT-MSCs in lung tissue samples of the SV group (8.0 ± 1.6 cells) was significantly less than that in the PV group (56.0 ± 16.1 cells) (*P* < 0.05) (Figure 9(g)).

### 3.5. Effects of AT-MSCs on mRNA Expression in Acute Hepatic Injury Induced by CCl_4_

#### 3.5.1. Proinflammatory Cytokines

**Figure 10:**
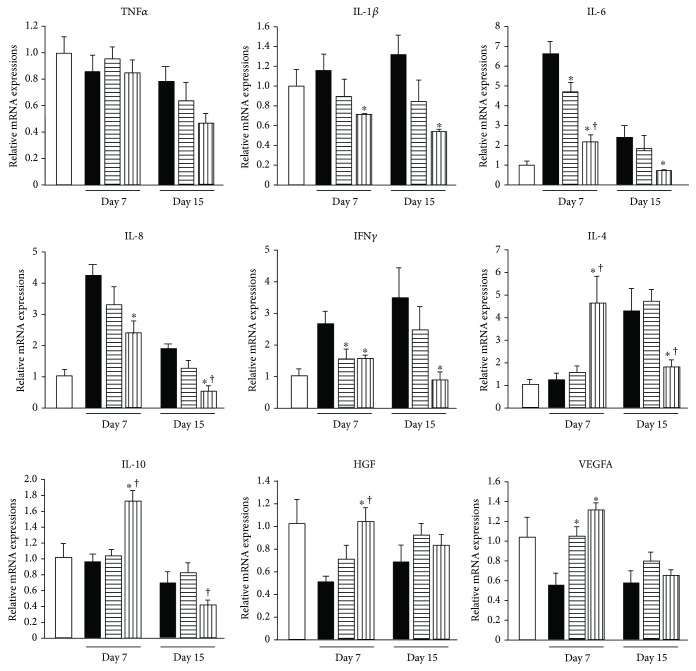
Comparison of mRNA expression in livers at day 7 and day 15. □: normal liver samples obtained from all nine dogs at 28 days before injection of CCl_4_, ■: control group; ▤: PV group; ▥: SV group. Data are expressed as the mean ± standard deviation. ^∗^*P* < 0.05 versus control group; ^†^*P* < 0.05 versus PV group.

TNF*α* expression was not different among the groups on days 7 and 15. However, IL-1*β*, IL-6, IL-8, and IFN*γ* mRNA expression was significantly decreased in the SV group compared with the control group on both days 7 and 15 (*P* < 0.05). IL-6 and TNF*α* mRNA expression on day 7 in the PV group was also decreased compared with the control group (*P* < 0.05) (Figure 10).

#### 3.5.2. Anti-Inflammatory Cytokines

mRNA expression of anti-inflammatory cytokines such as IL-4 and IL-10 mRNA expressions on day 7 was significantly increased in the SV group compared with control and PV groups (*P* < 0.05). IL-4 and IL-10 mRNA expression on day 15 in the SV group was also decreased compared with the PV group (*P* < 0.05) (Figure 10).

#### 3.5.3. HGF and VEGFA

HGF mRNA expression on day 7 in the SV group was increased compared with that in the control group (*P* < 0.05). VEGFA mRNA expression on day 7 in PV and SV groups was also increased compared with that in the control group (*P* < 0.05) (Figure 10).

## 4. Discussion

Our findings demonstrated that allogenic AT-MSCs ameliorated acute hepatic injury in dogs. In this study, the therapeutic effects of AT-MSCs based on the transplantation route were compared in canine acute hepatic injury induced by CCl_4_ injection. The serum biochemical parameters reflected hepatobiliary damage, such as markedly increased ALT, AST, and ALP levels. After the first AT-MSC injection, the levels of ALT and AST were significantly decreased in PV and SV groups. Moreover, the ALP level was also decreased in the SV group. In previous studies using rodents [12–15], similar results were obtained, including reductions in serum levels of ALT and AST after AT-MSC injection. Our results demonstrated that splenic vein and/or splenic parenchymal injection were predominantly effective for reductions of liver enzymes compared with peripheral injection. However, Nicolas et al. [1] examined the therapeutic effects of AT-MSCs on murine acute liver failure by comparing transplantation routes via the tail vein, portal vein, and liver parenchyma and obtained different results from our study. The previous study demonstrated that AT-MSC injection via the tail vein results in more prominent reductions of ALT and AST levels than via the other routes. These results suggest that AT-MSCs injected via the peripheral vein persisted relatively longer in the systemic circulation than AT-MSCs transplanted via the other routes. Therefore, the endocrine and/or paracrine effects of AT-MSCs were enhanced. It has been reported that AT-MSCs secrete several beneficial cytokines and growth factors. Recently, increasing data have shown that the therapeutic effects of AT-MSCs are achieved not only by their ability for differentiation but also paracrine release of cytokines and growth factors [16–18]. However, it is unclear whether factors secreted from AT-MSCs circulating in the systemic bloodstream or AT-MSCs engrafted in several organs contributed to the recovery of organ dysfunction.

Our results demonstrated that more AT-MSCs transplanted via the splenic vein or splenic parenchymal were engrafted in the liver compared with transplantation via the peripheral vein. By comparing AT-MSC uptake by specific organs including the lungs, liver, spleen, and bladder after transplantation into healthy beagles [19], it was shown that the systemic intravenous injection led to almost all AT-MSCs being trapped in the lungs, but high homogeneous diffuse hepatic uptake was observed via splenic injection. However, AT-MSCs have the ability to home toward injury sites [18]. Indeed, when AT-MSCs are transplanted into hepatic injured animals via the systemic route, AT-MSCs home to the injured liver [12, 20]. The mechanisms of AT-MSC homing have been researched and consist of various factors and signaling pathways [18]. Local injection is an invasive procedure that can also disrupt the highly complex and delicate microenvironment. Therefore, recent studies have preferred systemic injection as the delivery method of AT-MSCs. According to our results of canine acute hepatic injury, more AT-MSCs transplanted via systemic route were trapped in the lung compared with transplanted via the splenic route. This result was similar to the previous report using healthy beagles [19]. Although AT-MSCs have homing behaviour for the recruitment of AT-MSCs in the wound site, it is preferred that AT-MSCs would be injected via the splenic or portal routes when expecting more AT-MSCs are engrafted in hepatic injury site.

In our study, alterations of serum biochemical parameters such as ALT and AST were similar between PV and SV groups. However, the alterations of histological recovery and cytokine mRNA expression in the injured liver of the SV group were more effective than those in the PV group. Immnohistochemical and histopathological evaluation showed that hepatic lesions, such as inflammatory cell infiltration and hepatic fibrosis, were improved after AT-MSC injection. Moreover, the degree of hepatic lesions in the SV group was better than that in the PV group at both days 7 and 15. The alteration of cytokine mRNA expressions in the SV group showed that proinflammatory cytokines, such as IL-1, IL-6, IL-8, and IFN*γ*, were decreased on both days 7 and 15, and anti-inflammatory cytokines, such as IL-4 and IL-10, were more increased on day 7 compared with the PV group. Moreover, mRNA expression of HGF and VEGF, which promotes hepatocyte regeneration and proliferation, was higher in the SV group. These changes suggested that AT-MSC transplantation via the splenic vein induce better improvement than transplantation via systemic circulation. However, our study has focused on clarifying the expression of mRNAs, and further studies are needed to evaluate the protein expression levels and mechanistic pathways of these genes.

Some studies have shown that engrafted AT-MSCs differentiate into hepatocyte-like cells [15, 18], but it is unclear whether these hepatocyte-like cells have functions similar to those of native hepatocytes. We did not evaluate whether engrafted AT-MSCs in the liver differentiated into hepatocyte-like cells in this study. However, we believe that if engrafted AT-MSC can differentiate into hepatocytes, this property may benefit from improvement of acute hepatic injury. In this study, we evaluated the effects of allogenic AT-MSCs on acute hepatic injury during short period; therefore, further studies should address how engrafted AT-MSCs change in the long term including the differentiation and function. Although the number of engrafted AT-MSCs transplanted via the splenic vein and/or parenchymal was higher than via systemic injection, the total number of engrafted AT-MSC was low in the whole liver. In addition, considering the decreases in serum liver enzymes soon after AT-MSC injection, soluble factors secreted from AT-MSCs might mainly contribute to recovery of hepatic injury. From our results, AT-MSC therapy could contribute to the inhibition of inflammatory responses and followed by fibrosis in the acute hepatic injury in the short duration.

## 5. Conclusions

This study demonstrated that allogenic AT-MSCs may be effective to improve acute hepatic injury in dogs. Because the serum biochemical parameters, such as ALT and AST, were significantly decreased after AT-MSC injection, soluble factors secreted from AT-MSCs may act in recovery of acute hepatic injury. Our results also suggest that the transplantation via the splenic vein is more effective than transplantation via the peripheral vein. This finding was related to the number of engrafted AT-MSCs in the liver. However, further examinations including how many AT-MSCs used for transplantation is effective, how engrafted AT-MSCs change in the long period, and whether it is certain that the effect of improving hepatic injury is dependent on the number of transplanted AT-MSCs are needed for veterinary clinical application.

## Figures and Tables

**Table 1:** List of antibodies for cell surface markers used in the study.

Antibody	Clone	Isotype	Source
CD14-FITC	M5E2	Mouse IgG2a	BD PharMingen
CD29-PE	TS2/16	Mouse IgG1	BioLegend
CD34-PE	1H6	Mouse IgG1	R&D Systems
CD44-PE	IM7	Rat IgG2b	BioLegend
CD45-FITC	YKIX716.13	Rat IgG2b	eBioscience
CD90-PE	YKIX337.217	Rat IgG2b	eBioscience

**Table 2:** List of antibodies for inflammatory cell infiltration used in the study.

Antibody	Clone	Dilution	Antigen retrieval	Source
Myeloperoxidase	59A5	1 : 200	121°C for 20 min in citrate buffer, pH 6.0	Novocastra
CD163	AM-3K	1 : 100	Microwave for 5 min in citrate buffer, pH 2.0	TransGenic

**Table 3:** Primers used for real-time quantitative RT-PCR.

Gene		Sequence (5′-3′)	Length (bp)	Accession number
TNF*α*	For	ACCACACTCTTCTGCCTGCT	259	DQ923808
	Rev	GATAGTGCCGTCAGATGGGT		

IL-1*β*	For	ATGAGGGCATCCAGTTGCA	62	NM_001037971
	Rev	CAAGAGTCTGAGGCATTTCGTG		

IL-6	For	CTCTGCACTGAGAAAGGAGATG	132	NM_001003301
	Rev	CTTCCAATCTGGGTTCAATCA		

IL-8	For	TTGCCTTGGTCTCTTCTTTATTCC	67	NM_001003200
	Rev	AGCAAGCATCCTACCTCACAGAA		

IFN*γ*	For	GAAAAGGAGTCAGAATCTGTTTCGA	63	NM_001003174
	Rev	TAATGGTCATCCTGCCTGCA		

IL-4	For	TAGCACTCACCAGCACCTTTGT	119	AF239917
	Rev	TGCATGGAGCTGACTGTCAAG		

IL-10	For	ACTTTGATGACGATGAGCTATGGA	73	XM_014109407
	Rev	CCGTCGGCATTCCCATACT		

HGF	For	AAAGGAGATGAGAAACGCAAACAG	95	NM_001002964
	Rev	GGTATTACTGAAGCTTGCTAGGCC		

VEGFA	For	TCAGGACACTGCTGTACTTTGAGG	133	NM_001003175
	Rev	GGCTTGTCAGGAGCAAGTGAA		

GAPDH^a^	For	GATGGGCGTGAACCATGAG	131	NM_001003142
	Rev	TCATGAGGCCCTCCACGAT		

GUS^a^	For	CCTCCTGCCGTATTACCCTTG	117	NM_001003191
	Rev	TCTGGACGAAGTAACCCTTGG		

RPS5^a^	For	TCACTGGTGAGAACCCCCT	141	XM_533568
	Rev	CCTGATTCACACGGCGTAG		

^a^Reference gene. Glyceraldehyde 3-phosphate dehydrogenase (GAPDH) was identified as the most stable reference gene for normalization of relative mRNA concentration using the basic GeNorm visual application for Microsoft Excel (https://genorm.cmgg.be/).
